# Tunable liquid crystal multifocal microlens array

**DOI:** 10.1038/s41598-017-17688-1

**Published:** 2017-12-11

**Authors:** José Francisco Algorri, Noureddine Bennis, Virginia Urruchi, Przemek Morawiak, José Manuel Sánchez-Pena, Leszek R. Jaroszewicz

**Affiliations:** 1 Department of Electronic Technology, Carlos III University, Madrid, 28911 Spain; 2 New Technologies and Chemistry Faculty, Military University of Technology, Warsaw, 00-908 Poland

## Abstract

A novel liquid crystal microlens array with tunable multifocal capability, high optical power and fill-factor is proposed and experimentally demonstrated. A specific hole pattern design produces a multifocal array with only one voltage control. Three operations modes are possible, “Off”, “Tunable Multifocal” and “Unifocal”. The design is patterned in both substrates. Then, the substrates are arranged in symmetrical configuration. The result is a high optical power in comparison with typical hole patterned structures. Besides, it is proposed a hexagonal pattern that produces a high fill factor, specially indicated for some applications as Integral Imaging. The array has several useful characteristics for this type of application: tunability for the loss of resolution; multifocal for extended DOF; high fill factor for increase the number of views; and low power consumption for integration in portable devices. Moreover, the optical characteristics of the proposed device could bring new applications in other fields.

## Introduction

Research on liquid crystal (LC) lenses has gained an increasing interest in recent years. The low-weight, tunable focus, low power consumption and broad range of applications makes them unique with respect to other technologies. Since the first LC lens was proposed by Berreman *et al*.^1^ and Sato *et al*.^2^ several decades ago, a lot of different structures have came up. Some of the most important are, patterned electrodes^3,4^ Fresnel lenses^5^, modal control^6,7^ and immersed microlenses^8^. Another option is to modify the LC mixture itself. For example, polymer networks^9^, concentration redistribution^10^, carbon nanotubes^11^, blue-phase LC^12^, dual frequency LC^13^, tunable frequency^14^. This last option, is used to improve the switching time of the LC. Polymer networks and blue phases (a special cholesteric LC phase), have microsecond response time but the optical phase modulation is very limited. Dual frequency and tunable frequency improve both characteristics. Some recent applications of LC lenses include, auto-focusing systems and optical zoom systems^15^, pico-projection systems^16^, correction of defects in holographic projection systems^17^, photovoltaics^18^ or bio-optics (endoscopy^19^, ophthalmology^20,21^). Another important application is also 3D vision^22^. The research on LC lenses for autostereoscopic vision has produced several interesting results^23^. Liquid crystal lenses can be used as a replacement of conventional arrays in spatial multiplexing technique. This technique is based on classical optical phenomena as diffraction, reflection, refraction and occlusion in order to deviate the images to different positions in front of the display. Several technologies to produce this effect have been proposed. Among them, occlusive and refractive elements are the most used. Occlusive elements usually consists on masks containing vertical apertures that cover the light at certain angles. On the other hand, refractive elements are based on microlenses, with the advantage of more brightness, view angle and less crosstalk (depending on the microlens quality). These techniques, which are already used in commercial products, have not produced enough interest in the users. Some reasons are the reduced 3D content, low qualities (producing crosstalk) and the key problem of vergence-accommodation conflict (the adjustment of the eye focus). In order to avoid this problem, several solutions have been proposed. For example, increase the number of views or use full parallax (multi-view^24^ or Integral Imaging^25^). Integral imaging (InI) have both characteristics, several views and full parallax, making it the most promising technology for autostereoscopic displays and cameras. Gabriel M. Lippmann proposed this technique in 1908. He used an array of microlenses to collect the scene information in several tiny versions of the same one, each one with a different angle and position (similar to have a sampled hologram). The scene can be reproduced by using also microlenses, in this case the reproduction have full parallax and the cue of accommodation. This makes the system one of the best solutions to reproduce 3D images without visual strain and fatigue. Thanks to current developments on microlens manufacturing, this technique has been exploited recently with the commercialization of the first plenoptic camera^26^. These types of cameras have several advantages e.g. rendering images of several views, digital refocusing, depth estimation, 3D reconstruction, etc. Despite this, the use of fixed microlenses has several limitations due to the lack of tuning. In the literature, is possible to find several proposals to solve these problems, for example an extended Depth of Field (DOF) was proposed in^27^ by using a multifocal fixed lens. For the loss of resolution, switchable LC lenses were proposed in^28^. The DOF has been demonstrated to be tunable in^29^ and^30^. In^31^ LC microlenses were proposed in order to increase the viewing angle or field of view (FOV).

In this work, a novel tunable multifocal microlens array is proposed and experimentally demonstrated. The tunability is based on the properties of LC materials. Three operations modes are possible, OFF, tunable multifocal and unifocal. These modes can be controlled with only one low voltage signal. Among others, one possible application could be InI. The array has several useful characteristics for this type of application: tunability for the loss of resolution; multifocal for extended DOF; high fill factor for increase the number of views; and low power consumption for integration in portable devices. Moreover, the optical characteristics of this device could bring new applications in other fields

## Design and Structure

Conventional lenses have two physical characteristics that modify a wavefront trough them, the contrast between the refractive index of the lens and the surrounding medium, and the curvature of the interface between them. In the case of LC lenses, the medium is non-homogeneous, in consequence the wavefronts decrease its speed in the optically dense regions and are accelerated in areas of lower density. Because LC lenses are actually GRaded Index (GRIN) lenses, the estimation of the focal length, f_GRIN_, of the LC lenses is simple if the focusing of parallel rays is considered (Eq. 1),

1 $${f}_{GRIN}=\frac{{r}^{2}}{2\cdot t\cdot ({n}_{\max }-n(r))}$$

where *R* is the lens radius (half lens pitch), *t* is the thickness of the lens (thickness of the LC layer) and *n*_*max*_ − *n(r)* is the difference between the maximum refractive index, *n*_*max*_ (at the optical axis of each lens) and the refractive index at the position *r* (that is, at the edge of the lens). In order to change the focal length, two structural parameters can be modified, the radius and the thickness. In this work, several focal lenghts are created by varying the radius of each microlens. If more contrast ratio between focal lengths would be required, the thickness could be also modified (by depositing photoresist of different thicknesses).

**Figure 1:**
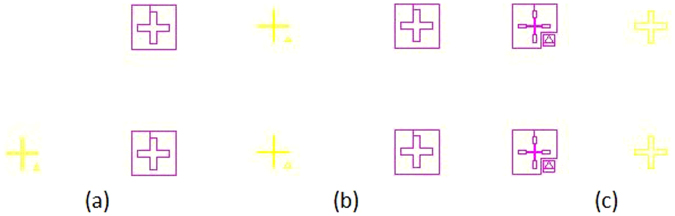
Sample of the used motif for align the two substrates.

**Figure 2:**
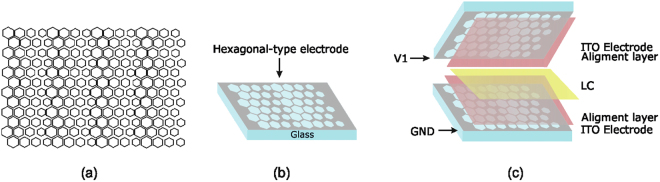
Tunable LC microlens array. (**a**) Hexagonal patterns for the mask, (**b**) depiction of one substrate, (**c**) depiction of the microlens array composition.

Symmetrical electrodes are used in order to increase the optical power, for this reason, special mask patterns have to be designed. Each square of the mask device is patterned with a specific motif (see Fig. 1),

The fabrication process requires a precise alignment of both upper and bottom substrates. Motifs of Fig. 1 allow alignments in the limit of the mask aligner. A mask aligner MA/BA6 Mask and Bond Aligner have been used. The MA/BA6 is equipped with a motorized top side alignment system that can reach an alignment accuracy of ± 0.5 µm. To reduce diffraction effects at the edge of the structured patterns, contact mode is used. In this process, the mask and the wafer are pressed all together. The same mask aligner has been used to align the top and the bottom hexagonal pattern before assembly the microlens device. Two ITO coated substrates (20 Ω/sq) with hexagonal electrodes patterned in each one Fig. 2(a) are prepared. This hexagonal aperture produces the hexagonal type microlenses. A polyimide (PIA2000) is deposited over the two substrates to have certain molecular alignment. Spacers of 40 µm diameter, mixed with optical glue, are deposited to separate the two substrates. Finally, a LC (E7) fills the cavity. The electro-optical parameters are: n_e_ = 1.7305; n_o_ = 1.5189 (Δn = 0.2116 at 633 nm, 25 °C)^32^. The diameters of the hexagons range from 120 to 90 µm in steps of 10 µm. The total active area of the array is 2.13 cm width and 1.42 cm height.

In order to have a high fill factor, microlenses with hexagonal apertures are designed. This allows a considerably reduction of the space between them. The symmetrical configuration produces a high optical power as it is demonstrated in the results. In comparison with typical hole lenses with the same aperture and thickness, the optical power is doubled. The use of symmetrical electrodes avoid the negative effect of a continuous ground electrode. When one substrate have 0 volts, the voltage distribution in most part of the LC material is below threshold voltage. For this reason, in asymmetrical configuration an insufficient use of the LC birefringence is made. The symmetrical electrodes produce voltages above the threshold voltage in the center of the microlenses. Thanks to this effect, the molecules are switched on in this region producing higher birefringence differences. In consequence, the phase difference with a symmetrical configuration is considerable higher. The main advantage of the proposed device is that only one voltage source is required. Moreover, low voltage signals are enough to switch the microlenses. Extended results are described in the next section.

## Experimental set-up and Results

**Figure 3:**
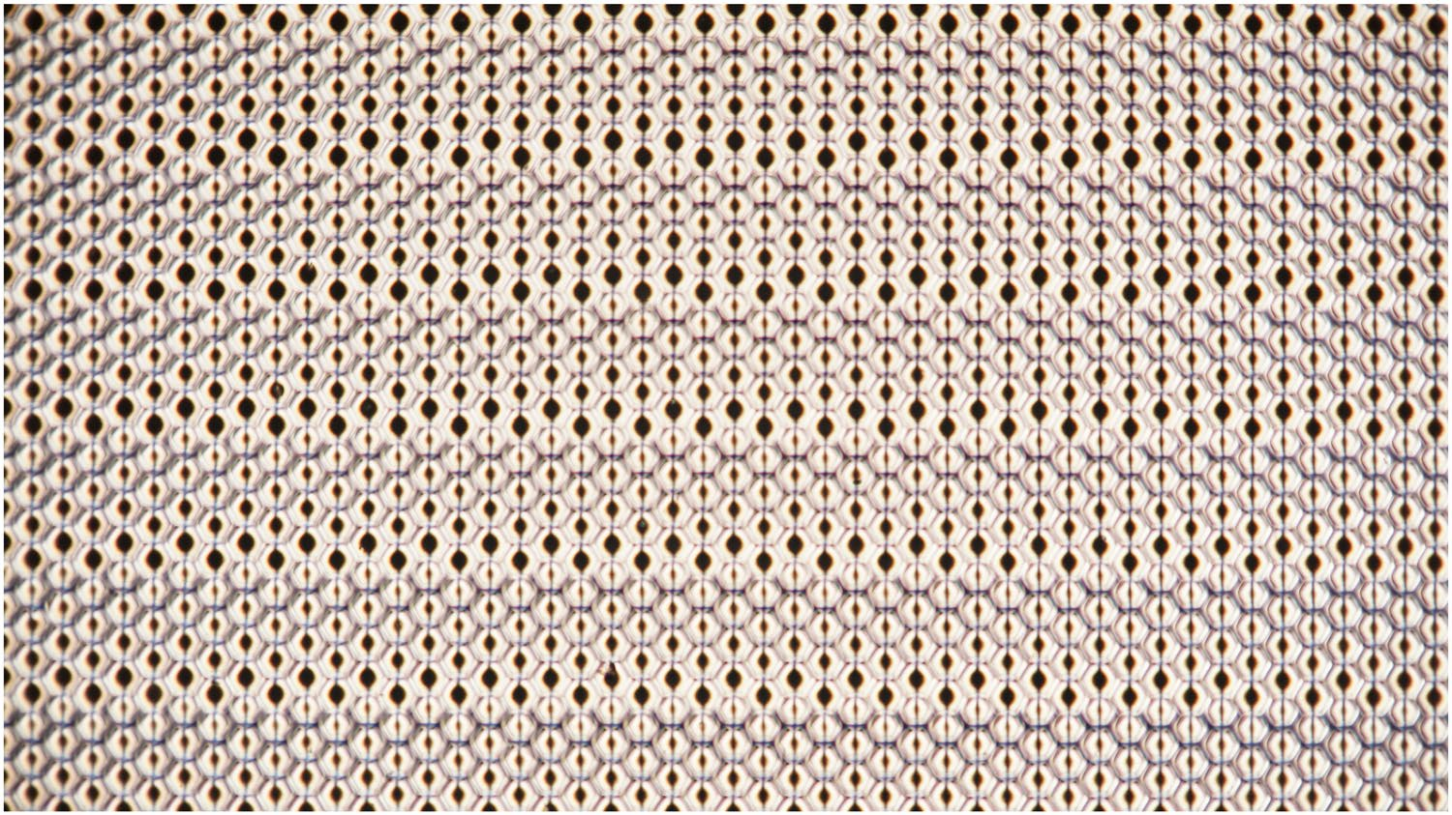
Experimental light intensity measurement by using a polarized microscope (×5). The applied voltage is 3 V_RMS_.

**Figure 4:**
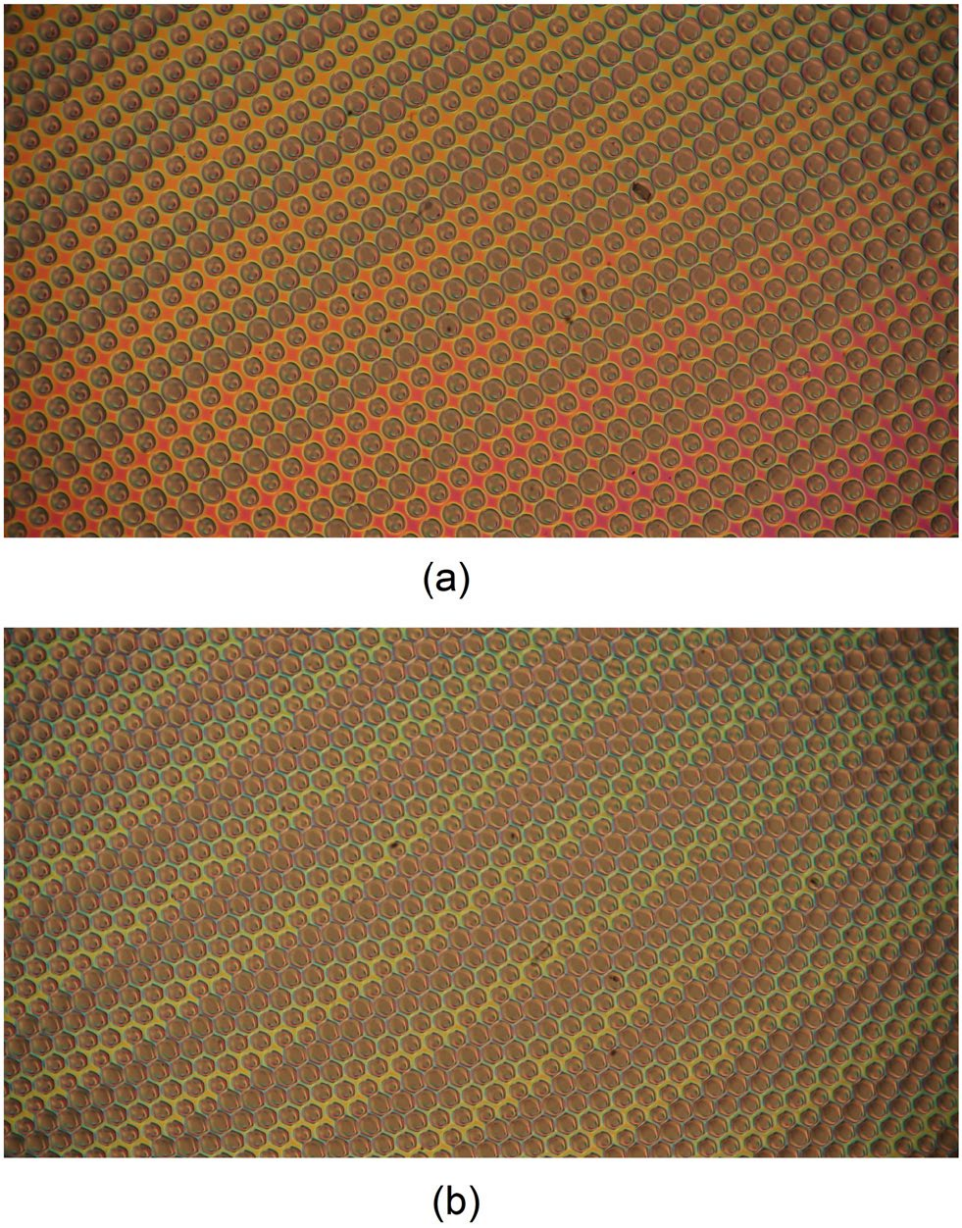
Experimental light intensity measurement between crossed polarizers and the sample at 45° for (**a**) circular apertures and (**b**) hexagonal. The applied voltage is 3 V_RMS_.

In order to demonstrate the homogeneity and the fill factor of the whole sample, Fig. 3 shows the switched sample by using a polarized microscope (x5 objective) and Fig. 4a comparison between the use of circular and hexagonal apertures.

The homogeneity of the sample is clearly stated. The fill factor is higher in the hexagonal pattern as the spaces between circles is avoided. The multifocal property can be observed through the different spot sizes. View that is more detailed is obtained through the fringe patterns. For this experiment, the system is placed in an optical table. The experimental set-up consists of: a He-Ne laser source (632.8 nm), a neutral density filter, a LC sample between crossed polarizers, a × 10 microscope objective and a B/W CCD digital camera (effective no. of pixels 1344 × 1024).

**Figure 5:**
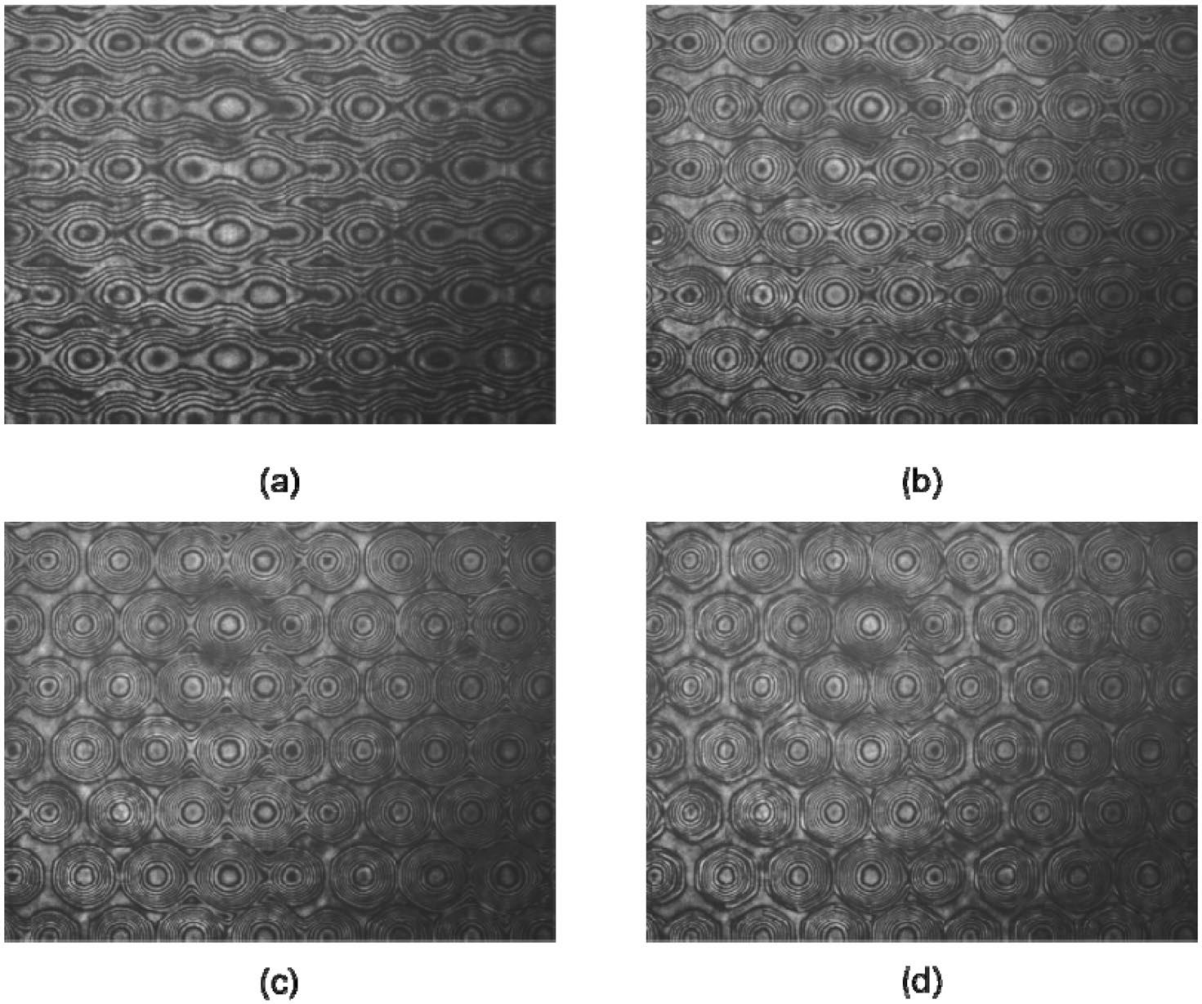
Experimental interference fringes patterns for different voltages, (**a**) 1.8 V_RMS_, (**b**) 2.2 V_RMS_, (**c**) 2.6 V_RMS_, (**d**) 2.8 V_RMS_.

**Figure 6:**
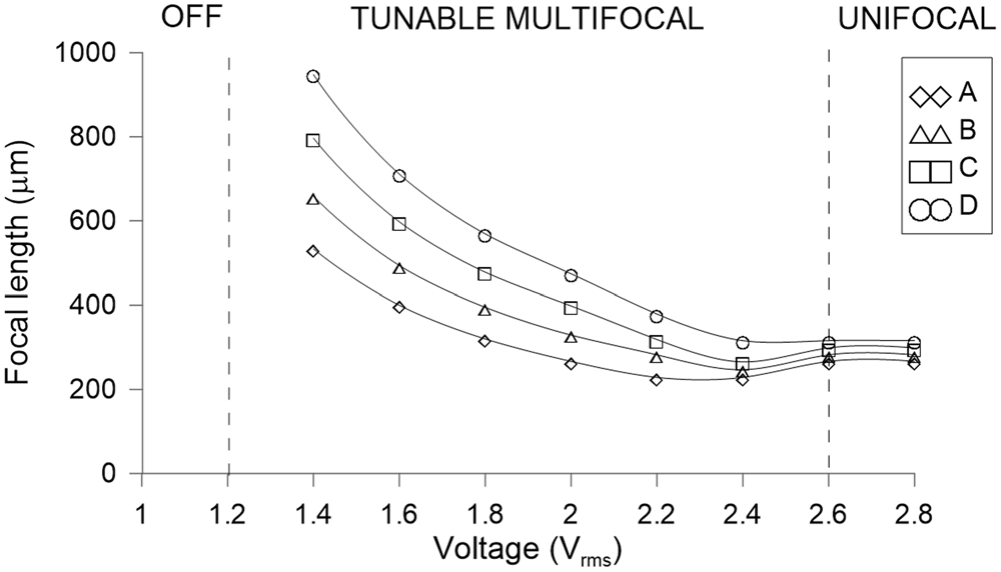
Resulting focal distances for microlenses with diameters of 120 µm (A), 110 µm (B), 100 µm (C) and 90 µm (D).

Figure 5 shows the experimental fringe patterns of the proposed device for several applied voltages. Every two bright fringes means an optical phase difference of 2π between them. Thanks to this, the phase profile and the focal length can be extracted from these results^33^. As can be observed, the lower the voltage the lower the number of fringes. This means that low optical power is obtained, generating a higher focal distance. From these observations three operations modes are expected. If the voltage do not reach 1.2 V_RMS_, the molecules are below threshold voltage and parallel to the substrate, in consequence there is not birefringence difference from the side to the center of the microlenses. This would be the “Off” mode. When the voltage is increased, the molecules start to switch in the sides, remaining parallel to the substrate in the center, and then the fringe patterns appear. Until 2.6 V_RMS_ the number of fringes is almost the same for the four type of microlens, 120 µm (A), 110 µm (B), 100 µm (C) and 90 µm (D). In this case, as the only parameter of Eq. 1 that changes is *r*, the focal distance changes in quadratic proportion. A multifocal tunable lens is obtained in this range (“Tunable Multifocal” mode). Moreover, the aperture is almost circular (see Fig. 4b and c). The elastic forces for these voltages are relatively low, the molecules have a smooth directional transition between them so the hexagonal aperture is almost circular. The last tunable range can be considered above 2.6 V_RMS_, two combined physical effects makes the lenses (A, B, C, D) to have the same focal length. Despite the radius is different, the number of fringes is also different. In this case, there is a limit of the maximum phase profile that can be obtained related to the ratio between the radius and thickness of the LC microlens^34^. The higher the radius the higher the birefringence difference. Both terms are offset against each other in Eq. 1, The result is a similar focal length in each microlens (“Unifocal” mode). In order to observe these effects, the focal length of each microlens for several voltages is plotted in Fig. 6.

As it was observed in the fringe patterns, there is a modulation region between the threshold voltage and 2.4 V_RMS_ (“Tunable Multifocal” mode). The maximum focal length ratio is 56% for 1.4 V_RMS_, with a focal length ranging from 948 µm to 533 µm. For voltages above 2.6 V_RMS_ the focal length, converge around 300 µm (“Unifocal” mode). As commented before, despite the radius is different, the number of fringes is also different so both terms compensate each other in Eq. 1.

In^31^ it was demonstrated a control of the FOV and the Multiview image processing with LC lenses. In this case, this mulifocal lens array would be capable of the DOF control with the added advantage of extension of the same one^27^. In addition, full resolution can be obtained in the OFF mode.

## Conclusions

A novel LC microlens array with multifocal capability, high optical power and almost full fill-factor has been proposed and experimentally demonstrated. The combination of a specific hole pattern design produces a multifocal array with only one voltage control. Three operations modes are possible, OFF, tunable multifocal and unifocal. Symmetrical electrodes are used. The result is a high optical power in comparison with typical hole patterned structures. Besides, it is proposed a hexagonal pattern that produces a high fill factor, specially indicated for some applications as Integral Imaging. The array has several useful characteristics for this type of application, tunability for the loss of resolution, multifocal for extended DOF, high fill factor for increase the number of views, and low power consumption for integration in portable devices. Moreover, the optical characteristics of this novel device could bring novel applications in other fields.
